# Fixed start method for repetitive project scheduling with simulated annealing

**DOI:** 10.1016/j.heliyon.2025.e41741

**Published:** 2025-01-07

**Authors:** Francisco Moreno, Eric Forcael, Francisco Orozco, Felipe Baesler, Duzgun Agdas

**Affiliations:** a College of Engineering, Universidad Panamericana, Guadalajara, Mexico; b Facultad de Ingeniería, Arquitectura y Diseño, Universidad San Sebastián, Santiago, Chile; c Engineering School of Sustainable Infrastructure and Environment, University of Florida, Gainesville, FL, United States

**Keywords:** Lines of balance, Variability, Discrete event simulation, Simulated annealing, Fixed starts

## Abstract

This research deals with the high project completion variability by presenting a new method to decrease such variability in repetitive construction projects. To achieve this, a Fixed Start Method (FSM) —where the starts of each activity with a high level of probabilistic confidence for the planned project duration are fixed— was applied, where the high level of probabilistic confidence obtained was optimized with the use of the metaheuristic algorithm called Simulated Annealing (SA). This procedure evaluated the project completion in a case study based on the coefficient of variance (COV) of the resulting standard deviation, mean, and temperature selected for the SA. Thus, the combined FSM and SA (FSSA) is a new method that decreases the variability of scheduling duration while achieving project completion times. Finally, the proposed FSSA method for construction scheduling was validated in a case study using Discrete Event Simulation.

## 1 Introduction

Delays are common problems in construction planning and scheduling; completing the project on time is crucial for avoiding additional issues on cost, time, contractors, and the owner [1], and consequences of project delay, reduction of profit margin, and the loss of confidence [[2], [3], [4]]. Viles et al. [5] determined that the three leading causes of construction project delay are problems during execution, administrative issues, and labor conflicts. As a result, early-stage planning touches on all these points, with which it can be assumed that working on planning with models beyond the traditional ones could help reduce delay times and conflicts and regain confidence in project deliveries.

With inherent complexity, the construction industry is incredibly susceptible to various unpredictable factors, and generating time delays is regarded as a critical factor of the adverse effects, bringing uncertainty in contractual terms and deadline compliance. The complexity and uncertainty of construction projects require simulation for analyzing and planning these projects when optimization can be used to review the inverse relationship between variability and duration of the projects [6]. Tools for predictive analysis of process outcomes, such as those developed in research using agent-based simulations, are lacking [7].

Similarly, variability in the production industry is a crucial aspect that produces uncertainty in terms of time completion, which is associated with the duration of the processes and also the lack of process flow [8]. In this sense, the Fixed Start Method was developed to help minimize the variability in time completion [9] and, consequently, enhance the project performance by considering a determined size buffer to be added to activity durations to increase the probability of maintaining the completion date of the baseline schedule, with the correct probability distribution function (PDF) [10]. Therefore, this research aims to optimize the buffer, minimize variability, and increase the efficiency of construction project completion times by implementing stochastic optimization algorithms.

Accordingly, due to their high variability, there is a permanent need for more confidence in delivering construction projects. Thus, the Fixed Start Method (FSM) has been developed to reduce the variability issues in completing repetitive projects in the construction industry, which considers expected durations based on significant confidence levels [9]. When searching for an interactive optimization, the person in charge of the decision-making process is used to supply information based on preferences while iterating up to reaching the most chosen solution [11] for the correct buffer size values affecting the variability and the delivery of the project with FSM.

Thus, this research consists of a method to schedule repetitive construction projects, which looks to minimize the variability of completion times by keeping the starts of each activity fixed, where the reliability of activities' duration is balanced by a time buffer, which gives more significant confidence levels for the expected times. Other authors have researched time buffers by size and probability density function (PDF) type for the activities [[12], [13], [14], [15]]. However, engineering problems that require optimization approaches are complex because they often consider multi-objective analyses with variables or loads that behave randomly [16]. In this case, the method considered in this research has been developed to deal with the job-shop scheduling problem aimed at reducing time by minimizing the variability using the Fixed Stat Method (FSM) and the Simulated Annealing (SA), which has been designated as “*Fixed Start with Simulation Annealing*” (FSSA).

The research objective is to reduce the variability in construction project completion using the simulated annealing tool in a fixed-start model (FSM). The previous model (FSM) developed by Moreno et al. [9] considered 95 % certainty to determine the planned duration. However, the present model uses SA to optimize the percentage of certainty by providing less variability and ensuring no interference between the work fronts while achieving the project completion on time. Thus, optimization of planning in the early stages, using FSM and SA (or FSSA), with which the optimal sizes of planned duration are determined, with the optimal crew sizes, and considering zero interference within the spaces, respecting the construction processes that will be carried out within the model to be evaluated in a case study, as the basis for the project's completion within the planned times.

Thus, the research contribution of using optimization tools with algorithms based on a simulation model annealed with a fixed start method is to solve interference conflicts between construction crews, optimize delivery times, and show how correct early planning helps contribute to the delivery of construction, meeting its complexities in repetitive construction projects. Based on this approach, it can be determined that variability and delays in project delivery can be mitigated with the FSSA model, considering optimal personnel management within the project.

## 2 Review of literature

### 2.1 Construction scheduling optimization

In the literature, diverse researchers address the problem of construction scheduling optimization. The most common approach involves evolutionary algorithms such as Genetic Algorithms (GA). In Xie et al. [17], the authors address a prefabricated building scheduling problem using GA. Another case utilizing GA is presented by Mathew et al. [18], where the authors solve the problem of scheduling repetitive projects to minimize project duration and cost. Additional examples of GA used as the primary approach can be found in Hassan et al. [19], Tran et al. [20], and Lazari et al. [21].

On the other hand, Li and Luo [22] and Tomczak and Jaśkowski [23] present a swarm intelligence-based approach. In these works, the authors employ Ant Colony Optimization and Particle Swarm Optimization models, respectively. It is also possible to find cases where mathematical modeling, particularly linear programming, is utilized. Two interesting examples employing this mathematical tool have been documented by García-Nieves et al. [24] and Monghasemi and Abdallah [25].

In this sense, Simulated Annealing (SA) emerges as a local search metaheuristic that can avoid local optima and continue searching toward global convergence. In most reported cases of SA in construction scheduling problems, this algorithm is integrated with other algorithms, typically GA. This approach creates a hybrid approach where part of the problem is solved by GA and another part using SA. Some examples of construction scheduling problems utilizing this approach have been developed by Bettemir and Sonmez [26] and Sroka et al. [27].

In the present article, the SA algorithm is used in its pure form to solve the problem of the Fixed Start Method for Repetitive Project Scheduling. This algorithm was chosen because it has proven to be very simple to implement compared to other metaheuristics. Moreover, it is a highly efficient approach, meaning it can find very good solutions in a short amount of time. In summary, its pure use is uncommon in construction scheduling problems, and its application to the specific problem of the Fixed Start Method for Repetitive Project Scheduling has not been reported.

### 2.2 Fundamentals of simulated annealing

One of the several simulation tools used for optimization problems is Simulated Annealing (SA), an extension of local search algorithms. It consists of a metaheuristic method to solve complex global optimization problems, where its objective function is not explicitly stated and assessed by using expensive simulation systems [28].

SA is a metaheuristic inspired by the annealing process in metallurgy, where a metal is slowly cooled until it reaches a low-energy state. The algorithm explores possible candidates, and in each iteration, it allows the acceptance of solutions that worsen the objective function. This decision to accept inferior solutions depends on the acceptance probability, which decreases as the metal cools. This cooling is controlled by a parameter called temperature, which decreases as the search process progresses. In the early stages of the search, the algorithm accepts many inferior solutions, but this gradually slows down as the search progresses, eventually becoming a local search. This intelligent mechanism helps avoid getting stuck in local optima, promoting a more global search for the optimal solutions. This method was introduced by Metropolis et al. [29], popularized by Kirkpatrick et al. [30], and has been used in several types of scheduling fields, such as machine scheduling [[31], [32], [33]], electrical networking [34,35], vehicle routing [36,37], among others.

In general terms, metaheuristic algorithms are an attractive alternative to managing incertitude problems and contribute to enhancing algorithms designed to optimize [38]. As part of this method of simulated annealing, within the iterative process, a starting significant θ value (probability annealing range) is found to be decreased. In this method, at the early iterations, a wire-ranging search within the space of solutions is produced, looking for convergence to the best solution that provides an adequate configuration disposing of the worst ones [39].

An unfavorable condition of the SA algorithm is the difficulty in controlling its factors, together with the annealing rate and starting temperature, where if its value is large enough, the algorithm has a higher chance of reaching optimal solutions [40]. However, simplicity is the most remarkable circumstance of SA since it is an analogy of material annealing that prevents Montecarlo's weaknesses (getting caught in local minimum values) because of a well-reached acceptance criterion of Metropolis [28]. In summary, SA is a metaheuristic, part of the methods based on local searches that provide progressive convergence into solutions close to optimal values [41].

### 2.3 SA in the construction industry

Uncertainty and variability in project completion within the construction industry have been analyzed for many years [[42], [43], [44], [45], [46]]. Many researchers have conducted diverse studies and investigations about the factors and causes of construction project delays, revealing that poor schedule performance in construction projects is identified as a comprehensive list of causes of delays, including ineffective or improper planning [47,48]. On the other hand, productivity and performance in construction projects are susceptible to the start of activities, mainly when diverse factors that conform to variability affect the project workflow [49]. In this sense, the need for appropriate initial planning emerges as a leading factor in high variability and delays [[50], [51], [52]].

In the construction field, SA has been used to optimize the management of some resources, such as crane management [53]; in the design, it has been used to generate spatial architectural layouts automatically from an architectural program specified by a user [54]; with the layout planning in temporary facility material layouts [55]; optimization of concrete frames with the use of SA, giving results in terms of constructability, cost, safety, and sustainability [56]; and determining time buffers and starts of activities considering the restriction of the expected deadline of the project [57].

### 2.4 Construction planning and scheduling methods

In general terms, significant research has been conducted over the last few years regarding scheduling. For example, some researchers have worked on elaborating a project portfolio selection and scheduling model while quantifying the dynamic synergetic effects to provide decision support for management [58,59]. Another case is developing a system that offers optimal scheduling decisions for mixed workloads in disaggregated data centers with theoretical guarantees, maximizing overall throughput while meeting latency service level objectives and decoupling the scheduling of different tasks [60]. Other researchers have developed predictive scheduling algorithms to balance machine utilization and order tardiness in steps [61] and face lot-sizing problems [62,63].

Regarding repetitive scheduling methods, for years, several research initiatives have been conducted within the construction industry, from some modifications of traditional scheduling techniques to sophisticated algorithms based on complex metaheuristics [64,65], as shown in Table 1. On the other hand, it is crucial to emphasize that when planning is carried out at the level of activities, it is feasible to ratify the allowable activity duration, considering the time-window constraints that emerge from the construction method and its relationships in terms of precedence [66]. In this sense, Hopp & Spearman [8] found two main factors that explain variability in processes when uncertainty is involved; the first one is the lack of flow in the planned activities, and the second is the variability in the duration of activities linked to the variability of the project.

**Table 1:** Research on scheduling methods and techniques in construction.

Authors	Scheduling methods and techniques in construction
Long & Ohsato [67]	Genetic algorithm for scheduling repetitive construction.
Hegazy & Kamarah [68]	Scheduling and cost optimization model for high-rise construction.
Jaśkowski et al. [69]	Discrete simulation evaluates feasible solutions (sequence of units) regarding schedule robustness.
Tomczak & Jaśkowski [70]	Harmonizing the work of crews that perform repetitive processes.
Altuwaim & El-Rayes [71]	Generate optimal tradeoffs between minimizing project duration and crew work interruptions.
Hassan et al. [72]	Optimizing the scheduling of crew deployments in repetitive construction projects while considering uncertainty in crew production rates.
Moreno et al. [9]	The method decreased the variability of scheduled duration while meeting project completion times.
Monghasemi & Abdallah [25]	The model provides new capabilities that enable planners to identify an optimal/near-optimal schedule that minimizes project total cost and the number of times crew work is interrupted.
Jaskowski & Biruk [73].	A mixed binary linear programming model can be solved using software available on the market or developed into a dedicated decision-support system.
Nguyen et al. [38]	In planning and estimating the project appropriately, uncertainty must be considered while implementing the project to represent a more realistic outcome for time, cost, and quality tradeoff (TCQT) problems in construction projects.
Bakry et al. [74]	Algorithm for schedule updating, dynamic rescheduling, and optimized acceleration of repetitive construction projects.

Regarding the duration of activities, this can be estimated in several ways by defining the activity duration (d) depending on its productivity, i.e., d=Q/P [75], where Q is the quantity of units of a task and P is the production per day of a workgroup or crew [76]. Methods such as PERT [77,78] use the central limit theorem, in which average and standard deviation of durations are estimated considering historical information. Here, the expectation is that the planned schedule will be directly impacted by the chance of 50 % of the project activities being delayed [79]. On the other hand, it is essential to keep in mind that the most utilized statistical distribution to explain how construction activities work is the beta probability density distribution (PDF) [12,13,80]; however, when high variability is found in activities (i.e., *COV* ranging from 100 to 150 %), Poshdar et al. [44] suggest considering the Burr statistical distribution.

Accordingly, a time buffer is a determined quantity of time that is summed up to the duration of an activity to shield such activity from the uncertainty and variability effects [81]. In this sense, to ensure the start of a subsequent activity is not affected, diverse authors consider adding a time buffer to the scheduled duration [14,75,82,83]. On the other hand, Khamooshi and Cioffi [84] worked with various completion probability percentages (ranging from 80 to 99 %) to evaluate variations in the behavior of the planned project. In other words, an extra resource that modifies an unbalanced transformation is considered a buffer, which can be classified as a time buffer (time-lapse that satisfies the difference between an expected and a real response in a construction procedure), a capacity buffer (additional transformation to meet the unexpected demand), and an inventory buffer (excess material in the conversion process) [8].

Finally, from the perspective of a production system, when a buffer is considered for machines, this buffer can modify the work sequence and hold jobs. To deal with this issue, Discrete Event Simulation (DES) computing programs have been used to model and simulate a production system that allows research on how work sequencing affects the reduction of production time [85].

## 3 Research methodology

The FSSA (Fixed Start with Simulation Annealing) method presented in this study considers a scheduled duration for every activity, where the completion confidence is high, considering fixed starts of the activities based on the original planning. The main characteristic consists of establishing fixed starts for every activity. At the same time, to reach on-time completion of the activities and, consequently, the project completion, it is necessary to set up a beginning project schedule, along with a planned duration that counts on a high confidence level.

**Fig. 1:**
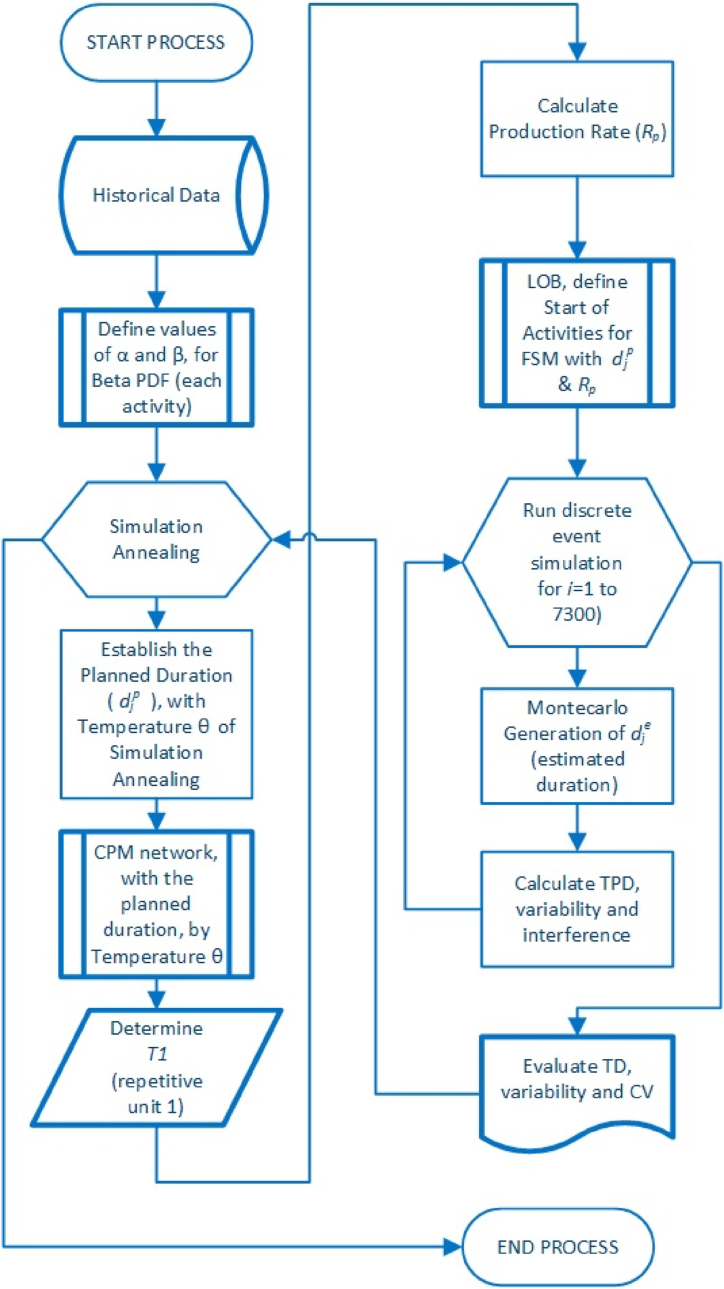
Fixed starts method with simulation annealing process diagram (adapted from Albor Consuegra & Dimitrakopoulos [86]).

Therefore, the stages to implement the FSSA method into projects that consider repetitive activities are graphically shown in Fig. 1. Consequently, according to what was established by Albor Consuegra & Dimitrakopoulos [86], the steps are described point by point in the following sections 3.1 to 3.9.

As seen in Fig. 1, the project's total duration (*TD*) as the objective function of this model can be identified. At the same time, the variables are all the activities of the repetitive type project (*j*), whose durations are determined from the estimated durations of the project (historical data), based on the PDF by activity, determining their alpha and beta data, respectively α and β. With these values, to model the system, the planned duration ($d_{j}^{p}$) is established from the % of accuracy expected for the model, determining the temperature to reduce the total duration (*TD*) and their variability (*COV*), and the variability in labor (*TPDD*, total of people per delivery day), based on the durations of the activities and having a precedence diagram of the project with their respective buffers. Once the temperature is established, the primary constraint when the model is run is that fixed starts will be kept constant throughout the simulation. Once the times are determined, an evaluation of the CPM model is carried out, and using a Monte Carlo, the FSM is compared with the traditional duration model. This temperature is then modified over and over, running the values and the SA process until the best temperature of the model is achieved, delivering the least variability with the shortest project completion time, thus optimizing the FSM model.

The size of the crews affects the time considered for the activities’ duration, but it is also required that there is no interference between the crews within the working space; they can cohabit within the space as long as the construction process and the space itself of the area are not affected. Several routes can be generated within the space so the personnel can work without interference.

### 3.1 Stage 1. determination of PDF per activity

According to previously recorded information, PDF will be determined for each activity, adjusting such data to the Beta distribution, which, as before mentioned, is appropriate to express how construction activities behave [12,13,44]. After this, the planned duration of each activity is calculated by adding a time buffer to the expected duration, allowing an extended amount of confidence to ensure that the planned duration is more or equal to the actual duration of each activity.

The traditional scheduling method determines the logical sequence of activities for the first repetitive unit, where a Precedence Diagram Method (PDM) and CPM are considered. Then, the first unit's start date, and consequently the completion time, are determined, —after the planned durations and network have been calculated—. Subsequently, using the Line of Balance (LOB) method [14,83,87,88], the production rate ($R_{p}$) is calculated to be applied to each activity in the project [76]. Accordingly, the fixed starts for each activity and their related planned completion are determined.

Before starting the simulation, based on historical data, it is required to identify the PDF for every activity and remove potential outliers. At that point, it is also necessary to set up the αandβ values of every activity (considering Beta distributions) through equation (1), with a gamma function Γ that ranges between 0 and 1, where historical data is standardized by establishing $x=\left\|d_{j}\right\|$ with ‖∙‖ representing the standardized vector.

(1) $$f\left(x;\;\alpha ,\beta \right)=\Gamma \left(\alpha +\beta \right){\Gamma \left(\alpha \right)}^{-1}{\Gamma \left(\beta \right)}^{-1}x^{\alpha -1}{\left(1-x\right)}^{\beta -1}$$

### 3.2 Stage 2. Definition of the percentage for PDM with Simulation Annealing

**Fig. 2:**
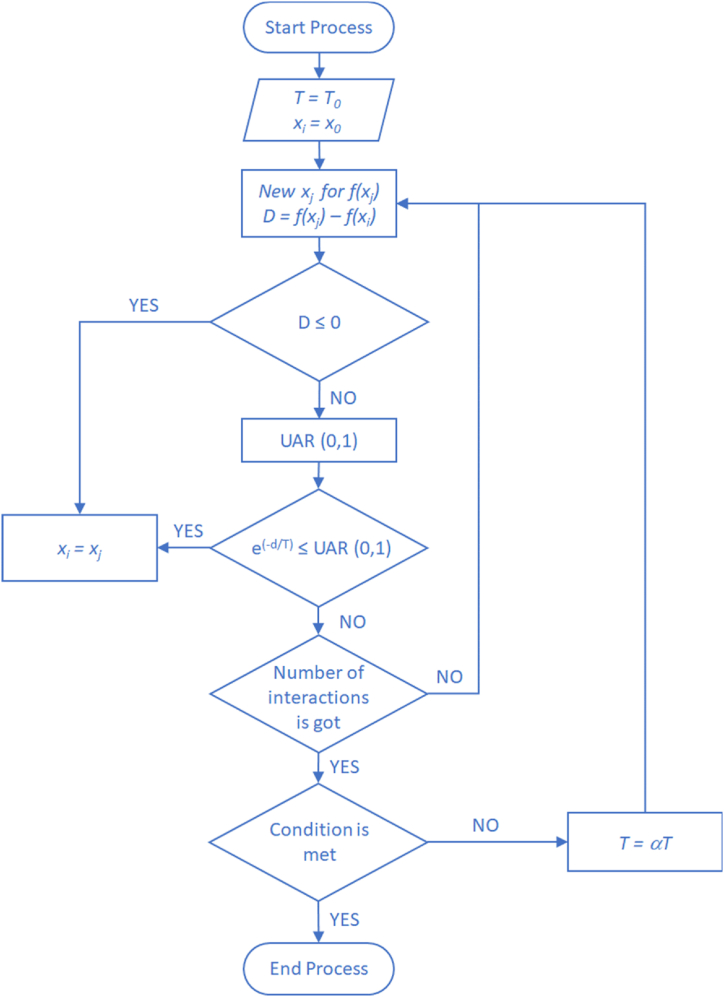
Flowchart of the SA algorithm, adapted from Zhou et al. [65].

First, the necessary adjustments were made to the general algorithm of the simulated annealing, showing its flowchart in Fig. 2, where it starts from an initial solution $x_{0}$ (higher value, generating $COV_{0}$) and a specific temperature $T_{0}$ (θ for percentage for planned duration PDM), chosen uniformly at random (UAR), *e ∼ U (*$\theta_{\min}$*,1)* in order not to affect FSM), and in each stage n in L iterations are performed.

The SA algorithm begins with a higher temperature —known as the initial temperature— gradually decreasing until the algorithm's local solution becomes steady. After that, seeking the global solution of the objective function, that local solution is jumped according to a determined probability [65]. In each iteration, a solution $x_{j}$ belonging to the space of neighboring solutions of the current solution $x_{i}$ (generating $COV_{i}$ for each $\theta_{i}$) is generated and is accepted as a new current solution through the application of the following probability of acceptance ($P_{T}$) as shown in equation (2) [30,65]:

(2) $$P_{T}\left(acepted\;x_{j}\right)≔\left\{ \begin{array}{cc} 1 & for\;f\left(x_{i}\right)\leq f\left(x_{j}\right)\\ \exp\left[\frac{f\left(i\right)-f\left(j\right)}{T}\right] & for\;f\left(x_{i}\right)>f\left(x_{j}\right) \end{array}\right.$$

After the last iteration, the temperature T(n)whereT=θ(n) for the average planned duration, the size buffer is decreased. Subsequently, the necessary adjustments were made to the simulated annealing algorithm, as seen in Fig. 2.

### 3.3 Stage 3. planned durations and CPM

**Fig. 3:**
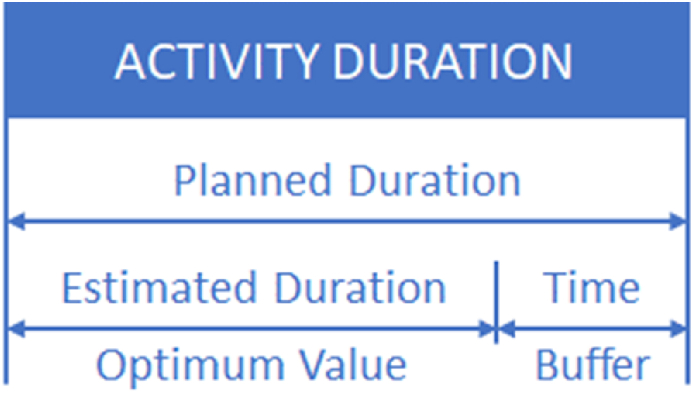
Activity Time Buffer (adapted from Russell et al. [81]).

Based on historical data for each activity, the α and β values will be defined to fit their corresponding Beta PDF [80,89]. Then, the planned duration ($d_{j}^{p}$) for every activity (j), is determined by using the law of Parkinson, which establishes that work expands to fill the time available for completion. On the other hand, Izmailov et al. [90] state that the duration of activities considers conjunctures, a fundamental principle of some probabilistic scheduling techniques [91]. To calculate the planned duration ($d_{j}^{p}$) for every activity (j), the entire value that covers at least a range of θ (probability annealing range) from 50 % to 99 % probability will be considered according to its PDF [86]. Consequently, a very high probability of completion for every activity is provided [84]. According to Lucko [14] and Ammar [83], equation (3) establishes that the planned duration ($d_{j}^{p}$) is the addition of $d_{j}^{e}$ (estimated duration) and ${bf}_{j}$ (time buffer for every activity), which is graphically represented through Fig. 3.

(3) $$d_{j}^{p}=d_{j}^{e}+{bf}_{j}$$

It has to be noted that the theorem of constraints places the buffer at the end of the project [9], whereas the FSM model places the buffer in each of the activities that are on the critical path, seeking to have the least interference between the models and generating a new planning model in addition to that proposed by Goldratt [92].

### 3.4 Stage 4. the planned duration of the first unit

Consequently, from the PDM, their corresponding CPM network, and the activity sequences and dependences [93], the time required to carry out the first repetitive unit (${TD}_{1}$) is obtained as the addition of activities’ planned durations ($d_{j}^{p}$) as shown in equation (4).

(4) $${TD}_{1}=\sum_{1}^{j}d_{j}^{p}$$

A CPM model is used, and the precedence diagram depends on the planned durations. For the FSSA model, this can be modified depending on the percentage of certainty of the planned duration, allowing the critical path to change between temperatures. In this way, it operates as a machine learning mechanism, learning from the CPM network and adapting to its operation.

### 3.5 Stage 5. production rate estimation

Then, the expected production rate ($R_{p}$) to complete the project is determined by adjusting the equation suggested by Ammar [83], excluding at the end of the project the time buffer considered in every activity based on what was stated by Nguyen et al. [15] and Nguyen & Chua [94]. The production rate is calculated with Equation (5) [75], where N are the repetitive units built during the project time, ${TD}_{1}$ is the time needed to finish one unit (days), and ${TD}_{p}$ is the total time for the project completion (days).

(5) $$R_{P}=\frac{N-1}{T_{p}-{TD}_{1}}$$

The inverse of $R_{p}$ is the interval days for an activity between repetitive units ($L_{1}$). To implement this step, this value —rounded to the minor integer— is considered for the expected project time not to be exceeded (equation (6)), bringing an adjusted production rate ($R_{a}$), which determines the fixed starts of activities of the project (equation (7)).

(6) $$L_{1}:=\lfloor \frac{1}{R_{p}}\rfloor$$

(7) $$R_{a}:=\frac{1}{L_{1}}$$

### 3.6 Stage 6. fixed starts definition by using LOB

After getting the times for the first unit of the activities, a dimension matrix (N×M) is created, with N being the repetitive units and M the activities in every unit, where the duration values per unit (i) are determined for every activity (j). Based on the $R_{a}$ (adjusted production rate), the new total time for the project ($TP_{e})$ is calculated with the equation $TP_{e}=\left(N-1\right)\;R_{a}+T_{1}$, which is derived from equation (5), where $T_{1}$ is the time to build a unit. Thus, the fixed starts of the activities (s) are calculated by using equation (8) as follows:

(8) $$s_{i.j}=\left(s_{1,j-1}+d_{j-1}^{p}+\left(i-1\right)\frac{1}{R_{a}}\right)$$

For all i, from 1 to n, and for all j, from 1 to m, where $d_{0}^{p}:=1$ and $s_{i,0}:=0$. For a matrix S of size n×m. To calculate planned completion ($f^{p}$) for each activity, equation (9) is defined.

(9) $$f_{i,j}^{p}:=s_{ij}+d_{i,j}^{p}-1$$

Therefore, let S and $F^{p}$ be matrices of dimension n×m with the start and finish for each repetitive unit of the project's activities, with their respective components $s_{i.j}$ and $f_{i,j}^{p}$.

### 3.7 Stage 7. scenario simulation and LOB preparation

After setting up the planned values, the proposed scheduling with fixed starts for the project is validated. Using historical data with the durations of activities, simulation of potential scenarios for repetitive projects is carried out to compare traditional methods with the project's performance [9]. The $d_{i,j}^{e}$ (estimated duration of every activity) is acquired by simulating each scenario, giving as a result equation (10)*,* where e is chosen uniformly at random (UAR), i.e., e∼U(0,1).

(10) $$d_{i,j}^{e}=\left[x_{e}∙\left\|x\right\|\right]$$

The $f^{e}$ (estimated completions) are obtained in every repetitive unit for every activity, from the planned fixed starts, by using equation (11):

(11) $$f_{i,j}^{e}=s_{ij}+d_{i,j}^{e}-1$$

Then, let $F^{e}$ be the matrix of dimension n×m with components $f_{i,j}^{e}$ for each repetitive unit in all activities estimated for completion within the project.

Based on the acquired information, the LOBs of the project were calculated and plotted for every repetitive unit, considering the activities’ completion and starts [14], along with the cumulative progress per activity to define when a task is in process in a day t for every i (repetitive unit). Next, to reach the start of units and completion day of a task, a function is needed. To do it, matrices are first created to collect data that will be used later, where the function ${sd}_{t,i}$ is generated in terms of the t time (days) as the slope of the LOB for the activity start j in the unit i (see equation (11)), through accumulating these values for each activity j generating the LOB of their starts. Equation (12) indicates with a 1 if the activity progresses at a given time t. Equation (13) represents, for every task j in the unit i, the estimated (unplanned) progress for a determined time t time. Then equation (14) represents the accumulated estimated date, which will have progress given by time *t*, according to duration.

(12) $${sd}_{{\left(t,i\right)}_{j}}^{p}≔\left\{ \begin{array}{cc} R_{a} & for\;s_{ij}\leq t\leq f_{i,j}^{p}\\ 0 & other\;case \end{array}\right.$$

(13) $${sd}_{{\left(t,i\right)}_{j}}^{e}≔\left\{ \begin{array}{cc} 1 & for\;s_{ij}\leq t\leq f_{i,j}^{e}\\ 0 & other\;case \end{array}\right.$$

(14) $${aed}_{{\left(t,i\right)}_{j}}^{e}≔\left\{ \begin{array}{cc} \frac{1}{d_{i,j}^{e}} & for\;s_{ij}\leq t\leq f_{i,j}^{e}\\ 0 & other\;case \end{array}\right.$$

In this way, the formula has m number of matrices ${SD}_{j}^{p}$ of dimension $TP_{e}\times n$. In the previously mentioned matrices, the components of every row are added; that is, $\sum_{i=1}^{n}{sd}_{{\left(t,i\right)}_{j}}^{p}$ for all t which give m vectors of dimension $TP_{e}\times 1$. The vectors are merged into a new matrix ${TSD}^{p}$ (total planned starting days) with $TP_{e}\times m$ dimension. Correspondingly, the matrix ${TSD}^{e}$ (total estimated starting days) is created for the matrix ${TAED}^{e}$ (total advance for the estimated duration, in days). For the matrix ${TED}^{e}$ (total estimated completion days), ${ed}_{t,i}$ is a function of t time, in days, that determines if a task j finishes in unit i, and is defined as shown in equation (15):

(15) $${ed}_{{\left(t,i\right)}_{j}}^{e}:=\left\{ \begin{array}{cc} 1 & for\;t\geq f_{i,j}^{e}\\ 0 & other\;case \end{array}\right.$$

The ${ATSD}^{p}$ (accumulated total planned start days) corresponds to the matrix of components obtained from equation (16). Similarly, the ${ATED}^{e}$ matrix (accumulated total estimated completion days) is created, along with the ${ATAED}^{e}$ (accumulative total advance for estimated duration days).

(16) $${atsd}_{t,i}^{p}:=\sum_{t=1}^{tpf}{tsd}_{t,i}^{p}$$

Then, np is a vector with m×1 dimension, consisting of the quantity of workforce for every task. Thus, the ${TPD}_{t}$ (total workforce a day) is calculated according to equation (17):

(17) $${TPD}_{t}:=\sum_{j=1}^{n}{{np}_{j}tsd}_{t,j}^{e}$$

At the end of the model, the LOB for ${ATSD}^{p}$, ${ATED}^{e}$, and ${ATAED}^{e}$ were plotted. The final part is to show how the labor requirement varies between days, plotting the total active labor per day, ${TPD}_{t}$.

### 3.8 Stage 8. variability measurement for the duration of the project

For quantifying variability, the coefficient of variation (*COV*) is computed (standard deviation divided by mean, measured in terms of time) [8]. Here, *COV* values lower than 0.75 mean low variability; values between 0.75 and 1.33 mean moderate variability, and *COV* values that exceed 1.33 mean high variability [8]. Regarding the dispersion of the data, the Coefficient of Dispersion (*COD*) used to measure data is deemed highly dispersed. It is considered above normal distribution behavior if its value exceeds 1 [95]. The ${ATAED}^{e}$ (variability of project completion times) is measured by utilizing the *COV* values established by Hopp & Spearman [8] and the $TPD_{t}$ (workforce variability) through considering the *COD* and *COV*.

### 3.9 Step 9. Simulation Annealing Verification

Finally, it has to be checked if the generation of the simulated reseating has produced the appropriate optimization response (Fig. 1); otherwise, a new calculation is generated from the adjusted temperature with the updated values T=α∙T [96], considering the operating range of the percentage accuracy size for the *PDF* in the *PDM*.

## 4 Case study

The case study is intended to test the optimization model presented in this research, with which the completion of projects can be ensured through the use of FSM and SA, i.e., FSSA, based on determining the planned duration, optimizing the temperature of the buffer size within the project, along with procuring that the interferences between crews and the delay times are optimized to a minimum by combining both optimization tools. The model test through this case study can be replicated in other types of repetitive projects.

One case study was carried out to validate the proposed FSSA method. Here, the traditional method's behavior, in which the PERT method is utilized to determine the size of the duration of the activities and, for the scheduling a CPM with lines of balance, where 7300 scenarios were simulated, according to the study carried out by Byrne [97], with a probability annealing range θ of 50 %–99 % probability of confidence.

### 4.1 Repetitive units with a lot size of one

The case study corresponds to a residential housing project in Guadalajara City, Mexico, consisting of 49 repetitive i units with 22 j activities in total, as shown in Table 2.

**Table 2:** List of activities for the case study.

Code	Activity Name
A1	Leveling, excavation, and backfill foundation.
A2	Leveling, anchoring castles, and cast foundation.
A3	Sewer, excavation, and piping PVC material.
A4	Construction of water tank underground.
A5	Filling and compaction
A6	Fabrication of brick walls and mason castle at the ground level.
A7	Formwork, steel reinforcement frame, and concrete pouring at the first level slab.
A8	Electrical installation in the first level slab.
A9	Hydraulic installation in the first level slab.
A10	Fabrication of brick walls and masonry castle on the first level.
A11	Formwork strip from the first level slab.
A12	Formwork, steel frame, and melt rooftop level slab.
A13	Electrical installation in the first level
A14	Parapets and plain concrete.
A15	Strip the formwork from the rooftop level slab.
A16	Interior, plumbing, and gas installation.
A17	Roof bricks and plaster of parapets.
A18	External supply for electrical installations.
A19	Finishing on restrooms
A20	Finishing facade and courtyard
A21	Concrete finishing on the floor indoors and outdoors.
A22	Gypsum finishing indoors.

**Fig. 4:**
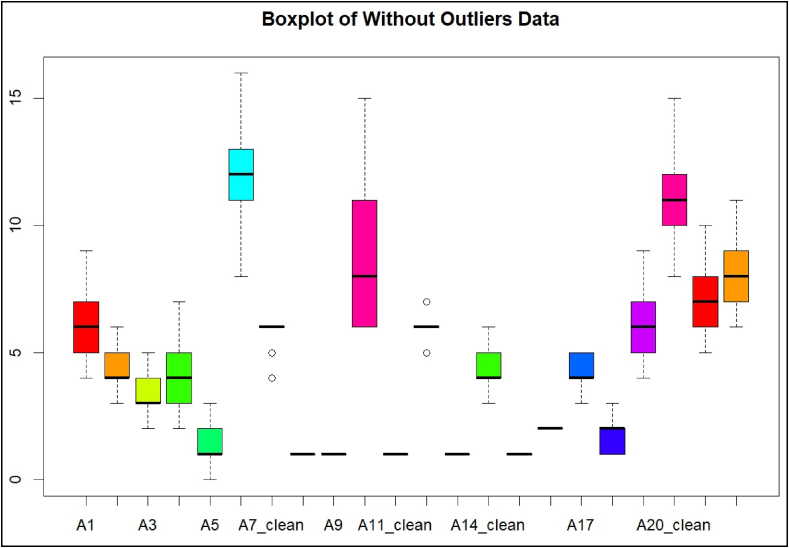
Activity duration without Outliers for activities 1 to 22 of the case study.

First, as previously mentioned, the historical values for each activity were considered, and the outliers were removed before proceeding with the simulation process. The debugged data was plotted in a boxplot graph, as shown in Fig. 4.

In terms of the simulation run, starting values were obtained and utilized in the fixed starts and traditional model for the $T_{p}=220$ days (the case study's original duration), and for the repetitive units of houses (N=50), with their respective activities (22 tasks).

**Fig. 5:**
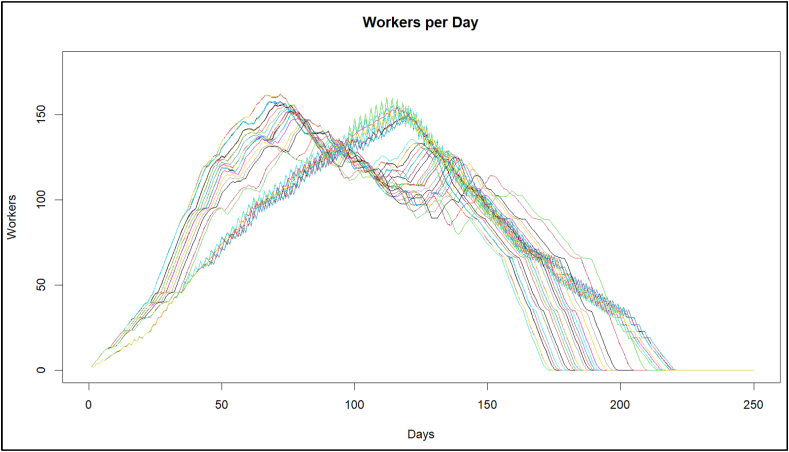
FSM Workers per day, with a range of 50 %–99 %.

Firstly, it was checked if the FSM model operates for any percentage value to determine the estimated duration, taking a range from 50 % to 99 %, as a pilot test, to start the simulated annealing based on the flowchart presented in Fig. 1 and which results are shown in Fig. 5, showing the variability and accomplishment of the last completion time, when FSM be on time for every average.

In Fig. 5, there are two ranges in which FSM stops operating correctly, it is the range from 50 % to 68 %, which occurs at these points; the transition between the black works and the finishes interferes with the activities, stopping FSM from working correctly due to the inference between the fronts, for which it tells us that if we have a range of less than 68 % in any other method, it will be unlikely that there is no inference between the activities.

**Fig. 6:**
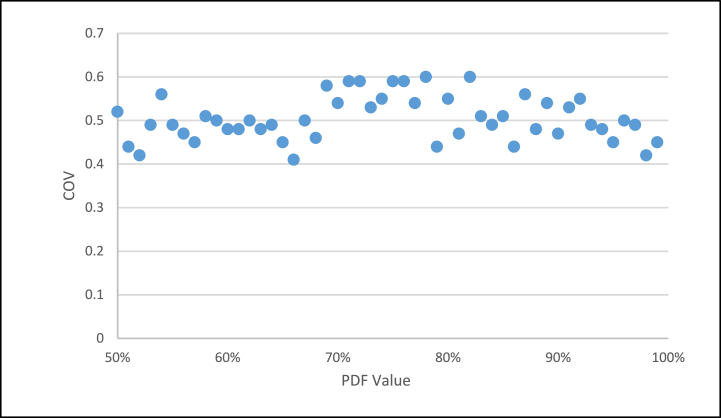
FSM, COV vs PDF Value.

With the values of each one of the results of the test, in which it was verified that both the *COV* and the total duration of the project have a better performance, with these values, and using the FSM model, the variability indexes were measured. The deliveries of the projects in the *COV* resulted in a high range of variability between 40 and 60 % (Fig. 6), which is within the range of low variability [8] and continuously significantly reducing the variability of the projects.

As observed in Fig. 6, the variability values of the project, observing the *COV*, it can be seen that these vary throughout the range, maintaining values between 0.4 and 0.6 of variability, for which before running the entire simulation, it was necessary to see the other variable that must be reviewed, which is the total duration of the project, and observe its delivery times.

**Fig. 7:**
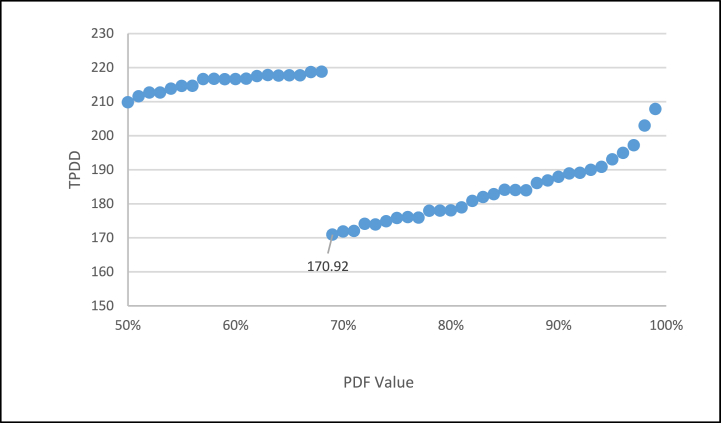
FSM, TPDD vs PDF Value.

Within this range, any results regarding the use of *COV* variability are the most positive. We must intervene in the second variant, which concerns the total duration of the *TPDD* (total project delivery days). Fig. 7 shows a behavior with a value before 68 %, showing a considerable increase in the *TPDD*. It can be observed that this indicates that FSM does not operate correctly in a range of less than 68 %. This finding is because the fixed starts and the ends of the predecessor activities must have interference, generating that the beginning of the activities must be displaced. In conjunction with Fig. 5, it can be determined that FSM does not operate correctly for values less than 68 % for the *PDF* of the activities.

**Fig. 8:**
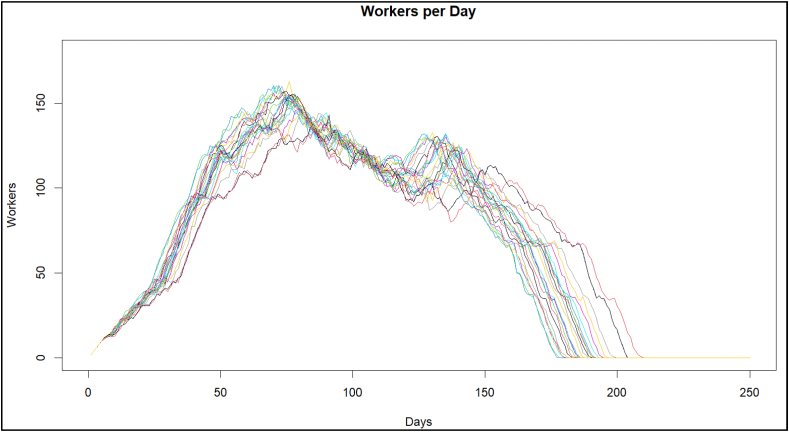
FSM Workers per day, with a range of 68 %–99 %.

It is observed in Fig. 8 that the curves that present two valleys within the image (the ranges from 50 % to 68 % were removed) are the ones with the best behavior. The increase in personnel caused by the finishing workers is opened within the construction, and there is no interference between structural and masonry works, and the finishing work causes the lengthening of the project deliveries. In this sense, FSM stops working correctly since buffers of the planned activities were lost, below the value of 68 % of the planned durations.

At this point, we know the range in which FSM works correctly for this case. However, it must add the variability that occurs in the *COV*, for which it was determined that the temperature for SA will be a combination of the form of a product between the total duration of the project and *COV*, with which both the reduction of the variability of the project is optimized. It is guaranteed to have the best project delivery time, making the complete optimization with SA using FSM and ensuring the best results for the construction project.

By having this review, it can be observed that the function to be reviewed by the simulated annealing should not only be the variability measured by the *COV*, in turn with the total duration of the project, to have a buffer at the end that can absorb the own variability in the operation of the FSM in repetitive projects, and with the CPM of the project.

It is observed that only the *COV* and the *TPDD* factors present different solutions in which a new product was added, which is the one generated between the multiplication of the *COV* with the *TPDD*, which gives a better solution because the *COV* can have an outstanding optimal value, but presenting a deterioration in the deliveries of the *TPDD* project, as observed in Fig. 6, Fig. 7 with the *PDF* value. The same situation happens with the *TPDD*, which has very short times with a high COV, so this product presents a solution to the variability of the project (*COV*) and its delivery time (*TPDD*) *(COV x TPDD*).

Accordingly, the data presented in Table 3 shows the behavior of the fixed start method with a variation in the percentage of certainty, ranging from 50 % to 99 %, where the changes of the *COV* in project deliveries, the *COV* of labor.

**Table 3:** Sample of SA for *COV* values times *TPDD* (total project delivery days).

	COV	DV	COV (labor)	TPDD	COV *x* TPDD		COV	DV	COV (labor)	TPDD	COV *x* TPDD
50 %	0.52	0.76	77.37	209.80	109.18	75 %	0.59	1.00	85.92	175.76	104.56
51 %	0.44	0.74	76.24	211.54	92.57	76 %	0.59	1.00	85.89	176.04	103.40
52 %	0.42	0.72	75.64	212.63	89.50	77 %	0.54	1.00	85.93	175.90	94.82
53 %	0.49	0.72	75.73	212.62	105.20	78 %	0.60	1.00	84.93	177.92	106.06
54 %	0.56	0.72	74.96	213.77	118.79	79 %	0.44	1.00	84.93	177.96	79.03
55 %	0.49	0.71	74.27	214.58	105.58	80 %	0.55	1.00	85.09	178.02	98.45
56 %	0.47	0.71	74.29	214.63	101.16	81 %	0.47	1.00	84.35	178.90	84.69
57 %	0.45	0.70	72.76	216.59	97.54	82 %	0.60	1.00	83.03	180.79	107.59
58 %	0.51	0.69	72.81	216.66	110.30	83 %	0.51	1.00	82.09	181.93	92.39
59 %	0.50	0.69	72.67	216.56	107.61	84 %	0.49	1.00	81.80	182.76	90.03
60 %	0.48	0.69	72.73	216.61	104.34	85 %	0.51	1.00	80.68	184.07	94.55
61 %	0.48	0.70	72.82	216.72	103.55	86 %	0.44	1.00	80.81	184.00	80.40
62 %	0.50	0.68	71.87	217.46	107.70	87 %	0.56	1.00	80.79	183.91	102.59
63 %	0.48	0.68	71.98	217.77	105.27	88 %	0.48	0.99	80.08	186.04	89.80
64 %	0.49	0.67	71.86	217.63	106.98	89 %	0.54	0.98	79.40	186.81	100.20
65 %	0.45	0.68	71.80	217.68	98.35	90 %	0.47	0.96	78.66	187.88	89.08
66 %	0.41	0.68	71.89	217.68	88.63	91 %	0.53	0.94	77.78	188.86	99.51
67 %	0.50	0.66	71.20	218.66	108.45	92 %	0.55	0.94	77.79	189.07	104.69
68 %	0.46	0.66	71.05	218.76	99.62	93 %	0.49	0.93	77.38	189.92	93.94
69 %	0.58	1.00	89.41	170.92	99.17	94 %	0.48	0.90	76.42	190.79	92.44
70 %	0.54	1.00	88.83	171.81	92.87	95 %	0.45	0.86	74.98	193.00	87.62
71 %	0.59	1.00	88.83	171.97	100.96	96 %	0.50	0.82	73.99	194.93	96.67
72 %	0.59	1.00	86.91	174.06	102.32	97 %	0.49	0.79	73.19	197.14	97.46
73 %	0.53	1.00	86.94	173.88	91.32	98 %	0.42	0.71	69.03	202.95	84.54
74 %	0.55	1.00	86.29	174.83	96.46	99 %	0.45	0.63	66.11	207.81	93.95

**Fig. 9:**
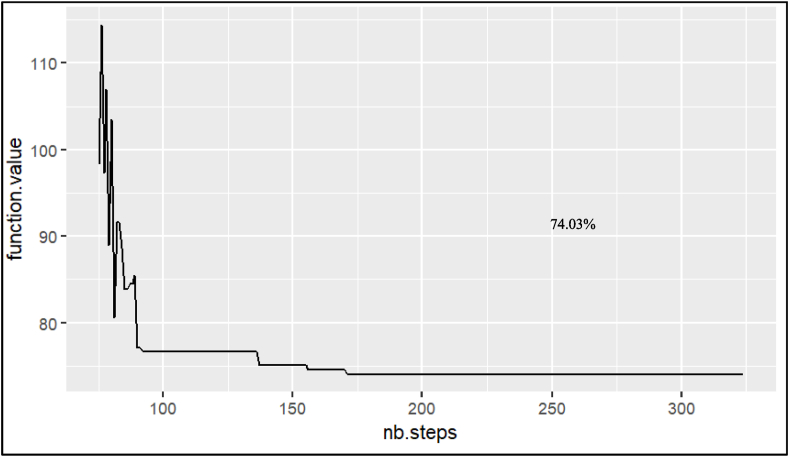
Optimization of temperature for FSSA

The simulated annealing algorithm's goal with FSM optimization is formed by two loops which are nested, generated by the Fixed Start method with Simulation Annealing (*FSSA*), giving both a fixed temperature and a solution; in each iteration, the internal loop generates a possible candidate solution subjected to an energetic assessment to evaluate if accepted or not as adequate [41], ensuring to have a global optimization metaheuristic. The mixed value of Variability (*COV*) and total duration of the project are shown in Fig. 9.

For its part, the individual optimization of variability and duration generated a problem in its equivalent variable; if the variability was optimized, it affected the duration of the project, and the same with time if the duration was optimized, it affected the variability; the solution was controlled by generating a product between the variability and the duration of the project, thus achieving *FSSA* optimization.

As the simulated annealing term, the values and the limits to optimize must be taken. The percentage of certainty used to take the estimated duration within the FSSA process will give the temperature value. In addition, we have *T*_*0*_ = 99 %, an optimized value with SA of 74.03 % percentage of certainty (Fig. 9); the value to be optimized is the total duration of the project (*TD*_*P*_) leaving the largest buffer size available with the current conditions according to the case study, with the values adjusted according to the *FSSA* and the value of the de (estimated duration, for the origin of the lines of balance with fixed starts), later entering the *FSSA* simulation model itself to determine the value of the variability in the deliveries of projects (*COV*), with the maturation of 1000 simulations between events.

Table 4 shows the simulation results for both approaches (*FSSA* with simulated annealing versus Traditional method), which considers the ${TD}_{1}$ (planned times for the first unit), $R_{a}$ (adjusted production rate), and $T_{p}$ (project total duration) —with 74.03 % of the optimal size of the activities explained before—, the number of days in the early project completion (when the project is finished after the expected date, negative values are found), the late completion percentage, the *COV* for delivery and workforce, and finally the *COD* for labor. Consequently, the findings are compared with the traditional CPM and PERT estimates [9].

**Table 4:** Case of simulation results FSSA versus traditional method.

Measurement Result	Unit	FSSA	Traditional
*T*_*P*_	days	220	220
*TD*_*1*_*Real (Average)*	days	121.75	130.09
*TD*_*P*_*Real (Maximum)*	days	177	213
*TD*_*P*_*Real (Minimum)*	days	117	100
COV (Delivery)		0.44	1.84
Days of early project completion (average)	days	42.11	6.25
Percentage of late completions	%	0.00 %	4.89 %
Standard deviation (Completion)		1.76	15.86
*COV* (Workforce)		85.03	76.18
*COD* (Workforce)		1	0.75

Table 4 also shows the final result of the selected temperature of the model and a comparison with the scheduling traditional method, where the durations are determined under a PERT approach and using the CPM diagram, and then compared with the FSSA model, which has durations based on the *PDF* of each activity and with the compliance of the SA procedure, whose durations and models are later analyzed in detail.

**Fig. 10:**
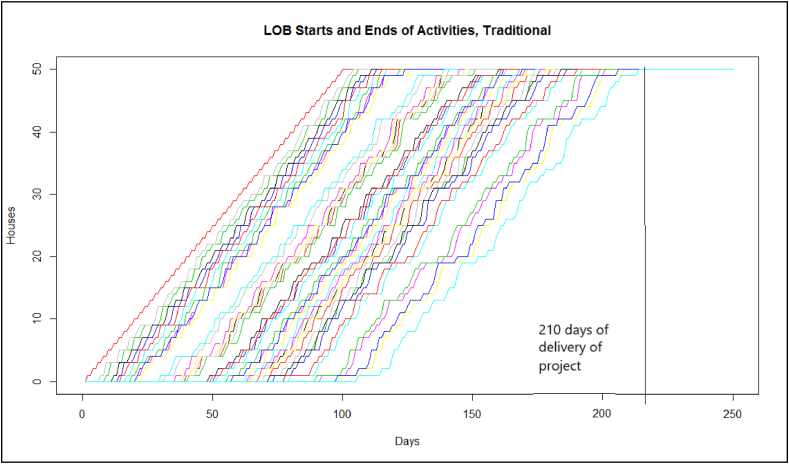
LOB for the traditional method (CPM and PERT) for the case study.

**Fig. 11:**
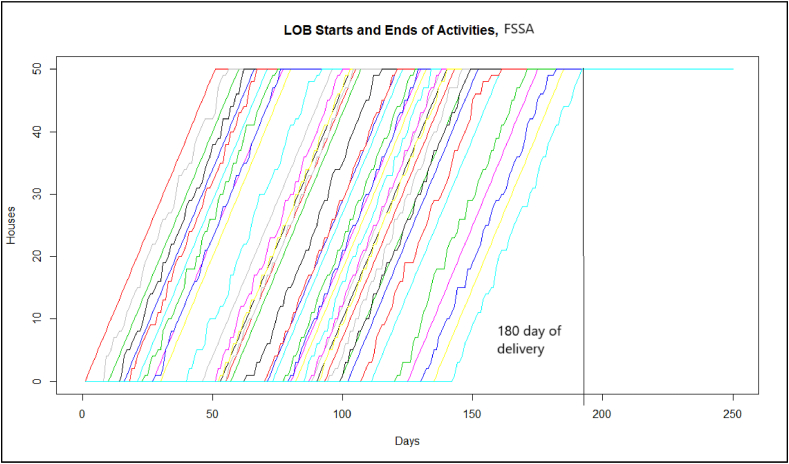
LOB for the Fixed Starts method with Simulated Annealing (FSSA).

In addition to the outcomes obtained by the DES, diagrams of LOB for the traditional method (Fig. 10) and for the *FSSA* (Fig. 11) were created. Both figures show the slopes obtained and the project completion times by each method.

**Fig. 12:**
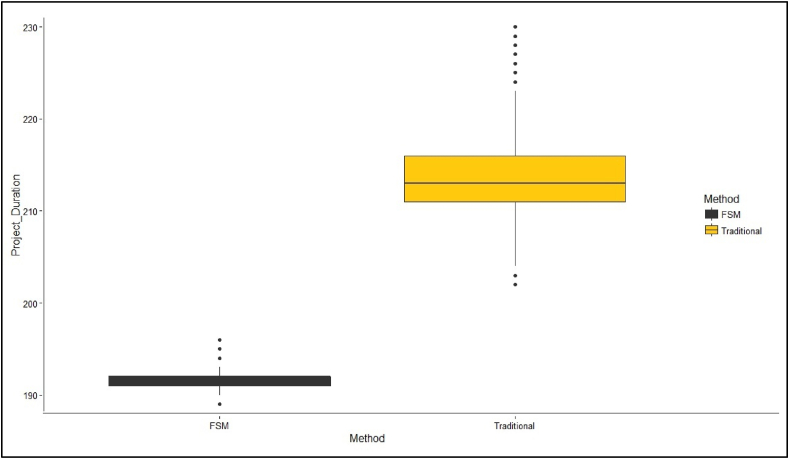
Graph with the project duration ranges, comparing the FSSA and traditional method.

Consequently, Fig. 12 shows simulations for both methods and the project's corresponding completion times. Thus, the *FSSA* may contribute to reducing the variability of project completion. The value generated by SA (74.03 %) shows how the variability behaves from the *FSSA* model against the traditional method, where it is seen that the behavior of the variability of project delivery, which is observed in the boxplot, how the project delivery dates are concentrated, showing the behavior of the *COV* of the project and, as with the traditional method, significant variability is observed in the total project deliveries.

### 4.2 Analysis of results

The present research shows the development of the SA algorithm and its application to the *FSSA* method. Specifically, once the change in the range of the estimated duration is implemented from the *PDF* (shown in Fig. 5), it stops to review the *COV* (as seen in Fig. 6) from this same percentage of certainty of the estimated duration of the *PDF*, after which it is observed that the *TDPP* has two approximation ranges; before 68 % with an increase in the duration of the *TDPP* project, and the range from 68 % to 100 % (Fig. 7), in which a much better duration is obtained, leaving the LOB within this same range from 68 % to 100 % (Fig. 8).

With this data, an optimized output is reached, in which the *COV* result is factored with the project's *TDPP*, optimizing the range. The *FSSA* is shown in Fig. 9, where the optimization of the *COV* product with *TDPP* is determined, which is a summary of the operation of the process optimization.

**Fig. 13:**
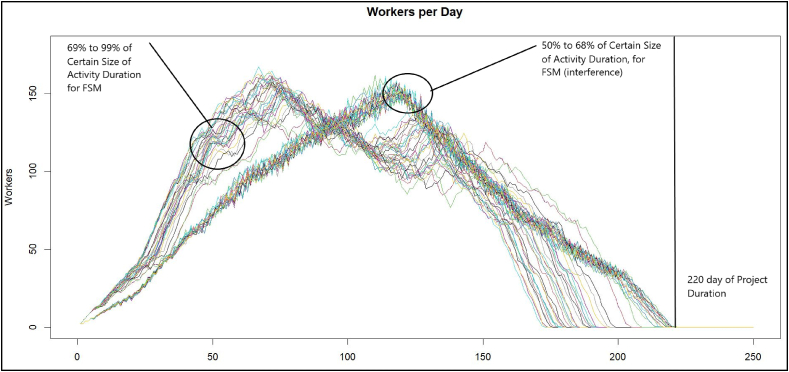
FSSA workers per day, with a range of reviews.

It is observed that the FSSA does not work with all the average of certainty for activity, in the range of 50 %–68 %, as seen in Fig. 13, presenting interference between the lines of the LOB chart and not generating the correct buffer size at the delivery of project, increasing the variability of the labor per day, the *FSSA*, have a delay and works directly as a CPM (where there is no warranty to accomplish project delivery on the date of time).

It is also observed that FSSA stops working if there is not an adequate buffer to guarantee the delivery of the projects. Based on the range lower values than 68 %, a delay in deliveries is observed, and the *FSSA* model stops operating properly (two standard deviation values), with which it was necessary to determine that this range should not be considered for the use of *FSSA*.

When carrying out the *FSSA*, the optimizations could be observed from two points of view: the first from the variability, as observed in Table 3, and the second data from its duration, so that taking the product of the *COV*_*j*_ *x TP*_*j*_ has allowed optimizing the value in terms of deliveries within a controlled range, generating a larger buffer in deliveries, which is capable of absorbing any variability that occurs in the deliveries of the projects.

In Fig. 10, Fig. 11, it can be seen how the *FSSA* with the lines of balance helps have deliveries on time and that the workforce is available once their activities are finished, giving continuity to the workforce, and with Fig. 13 in which deliveries are reaffirmed with a controlled variability range and with 100 % compliance.

As seen in Fig. 12, the simulated annealing simulation began using a change of variables using a Monte Carlo. By adopting the models within the *FSSA* process, it was possible to determine the appropriate size of the buffer by measuring two variables, variability and the duration of the projects, through which it was possible to optimize the size of the buffers. In turn, excellent efficiency was observed in project deliveries, meeting the goals.

Subsequently, the measurement of the *FSSA* process optimized with the traditional model was carried out, in which the results were reflected with the behavior of the LOB and the assurance of the *COV* of the project and by guaranteeing 100 % of the deliveries on time and form, managing to improve the *FSSA* process making it more efficient in its behavior.

A model that is based on the balance lines, whose main objective is to give continuity to work, has managed to be implemented to reduce the variability with the *FSSA*, guaranteeing deliveries in time and form, which is compared with Traditional planning methods, this model has been improved by optimizing the simulated annealing processes, which touches on the fundamental variables presented by the *FSSA* model, which is to have the continuity of the works and that they do not change in their beginnings to guarantee deliveries, this through a correct size of planned duration, this same, has been optimized employing the size of the buffer, obtaining for the case study a value of 74.08 %, with which we guarantee the deliveries of the projects on time, and the correct optimization of the variability of the deliveries, ensuring that the planning agreed at the beginning of the projects is fulfilled.

## 5 Conclusions

The present research shows that optimizing tools (SA) and the Fixed Start Method (*FSSA*) can solve conflicts among construction crews, enhance delivery times, and highlight the importance of early planning in repetitive construction projects. The *FSSA* model can help reduce variability and delays in project delivery by promoting optimal personnel management.

The use of the *FSSA* has contributed to ensuring deliveries by reducing variability, helping that the objectives are met in a more sustained way with the values of the expected durations depending on the size of the buffers, and this has been achieved through the use of the methodology of simulated annealing, which generates a whole opening to the optimization of processes within the construction, to guarantee the operation of models of repetitive and non-repetitive use, which can be seen in other investigations, applying a construction process optimization model.

The *FSSA* model can be measured by using other optimization algorithms, such as the Ant Colony Optimization method, in which one would seek to evaluate whether the original planning guides the correct path. With a Simulated Annealing by duration range of the activities, it could be possible to increase the multivariate by having different buffer behaviors, depending on the size of the activity's duration, with a change of trajectory within the CPM planning, achieving a more precise optimization, not only in repetitive processes but also in the planning of non-repetitive projects, where the *FSSA* could be implemented at the milestones or commitment dates of project delivery.

Using metaheuristic tools for multivariate calculation has been beneficial in determining the optimal buffer size for the project considered. However, further research can be conducted using metaheuristic tools combined with scheduling methods —such as *FSSA* presented here— but applied to other projects beyond building. In this sense, it should be noted that for different implementations, the appropriate values for the *FSSA* must be found, and the beta distribution must be determined if it is the most suitable or if new types of distributions should be proposed.

## CRediT authorship contribution statement

**Francisco Moreno:** Writing – review & editing, Writing – original draft, Visualization, Software, Methodology, Investigation, Formal analysis, Data curation, Conceptualization. **Eric Forcael:** Writing – review & editing, Writing – original draft, Visualization, Validation, Supervision, Project administration, Methodology, Investigation, Funding acquisition, Conceptualization. **Francisco Orozco:** Writing – review & editing, Validation, Supervision, Resources, Project administration, Conceptualization. **Felipe Baesler:** Writing – review & editing, Visualization, Validation, Supervision, Software, Methodology. **Duzgun Agdas:** Writing – review & editing, Writing – original draft, Visualization, Validation, Investigation, Formal analysis.

## Data availability statement

Data will be made available on request to the corresponding author.

## Funding

This publication was supported by the Vicerrectoría de Investigación y Doctorados de la Universidad San Sebastián, Chile —Fund number USS-FIN-25-APCS-01.

## Declaration of competing interest

The authors declare that they have no known competing financial interests or personal relationships that could have appeared to influence the work reported in this paper.

## Appendix – Notation List

**The following symbols are used in this paper**
${ATSD}^{p}$ =: Accumulated total planned start days
${atsd}_{t,i}^{p}$ =: Accumulated total planned start days per unit (i) in function of time (t)
${ATED}^{e}$ =: Accumulated total estimated ending days
${atsd}_{t,i}^{e}$ =: Accumulated total estimated start days per unit (i) in function of time (t)
${ATAED}^{e}$ =: Accumulative total advance for estimated duration
${bf}_{j}$ =: Time Buffer for each activity (j)
d =: Duration of activity
$d_{j}^{e}$ =: Estimated duration for each activity (j)
$d_{j}^{p}$ =: Planned duration for each activity (j)
${ed}_{t,i}$ =: Indicator of ends per unit (i) and activity (j) in function of time (t)
$F^{e}$ =: Estimated finish of units and activities
$f_{i,j}^{e}$ =: Estimated finish of unit (i) and activity (j)
$F^{p}$ =: Planned finish of units and activities
$f_{i,j}^{p}$ =: Planned finish of unit (i) and activity (j)
i =: Units
j =: Activities
M =: Number of activities
N =: Number of repetitive units
${np}_{j}$ =: Number of personnel per activity (j), crew elements
P =: Production of a working crew
$P_{T}$ =: probability of acceptance
$L_{i}$ =: Lapse of days between the beginning of units for the same activities
Q =: Amount of work in units per activity
T =: Temperature of simulation annealing
$T_{1}$ =: Total time of the first repetitive unit
$T_{p}$ =: Desired total time of the project
$TP_{e}$ =: Estimate total time of the project
$R_{a}$ =: Adjusted production rate
$R_{p}$ =: Production rate
S =: Start of units and activities
$s_{i,j}$ =: Start of unit (i) and activity (j)
${SD}_{j}^{p}$ =: Adjusted production rate planned per activity
${sd}_{t,i}$ =: Adjusted production rate of unit (i) in function of time (t)
t =: Time;
${TAED}^{e}$ =: Total advance for estimated duration (days)
${TPD}_{t}$ =: Total personnel or labor per day
${TSD}^{e}$ =: Total estimated starting day
${tsd}_{t,j}^{e}$ =: Total estimated starting day, per unit (j) in function of time (t)
${TSD}^{p}$ =: Total planned starting day; and
α & β =: Shape parameter (Beta Distribution)
